# Methylation of miR-129-5p CpG island modulates multi-drug resistance in gastric cancer by targeting ABC transporters

**DOI:** 10.18632/oncotarget.2594

**Published:** 2014-10-18

**Authors:** Qiong Wu, Zhiping Yang, Lin Xia, Yongzhan Nie, Kaichun Wu, Yongquan Shi, Daiming Fan

**Affiliations:** ^1^ State Key Laboratory of Cancer Biology and Xijing Hospital of Digestive Diseases, Xijing Hospital, Fourth Military Medical University, Xi'an, China

**Keywords:** MiR-129-5p, Gastric cancer, Drug resistance, ABC transporters

## Abstract

Recent studies have reported that hyper-methylation in the promoter region of miRNAs could silence the expression of tumor suppressive miRNAs and might play significant roles in the process of tumor development. However, the potential mechanisms regarding how methylation of miRNA CpG Island could regulate cancer cell chemo-resistance have not yet been studied. Using microarray and BSP (Bisulfate Sequencing PCR) assays, we found that compared with the parent SGC7901/VCR cells, expression of miR-129-5p was restored in SGC7901/VCR gastric cancer multi-drug resistant cell line treated by de-methylation reagent (5-AZA-dC). Using gain or loss of function assays, we found the over-expressed miR-129-5p reduced the chemo-resistance of SGC7901/VCR and SGC7901/ADR cells, while down-regulation of miR-129-5p had an opposite effect. Furthermore, three members of multi-drug resistance (MDR) related ABC transporters (ABCB1, ABCC5 and ABCG1) were found to be direct targets of miR-129-5p using bioinformatics analysis and report gene assays. The present study indicated that hyper-methylation of miR-129-5p CpG island might play important roles in the development of gastric cancer chemo-resistance by targeting MDR related ABC transporters and might be used as a potential therapeutic target in preventing the chemo-resistance of gastric cancer.

## INTRODUCTION

MicroRNAs (miRNAs) are small, non-coding RNAs with approximately 19~24 nucleotides in length. MicroRNAs can regulate expression of multiple targeted genes by inducing translational silencing or causing degradation of the targets through acting in association with the RNA-induced silencing complex (RISC) [1, 2]. It has been reported that miRNAs can regulate many malignant phenotypes of cancer, such as cancer cell proliferation, apoptosis, MDR, cell migration and invasion [3, 4]. Understanding miRNA functions and the potential malignant mechanisms requires elucidation of the molecular pathways that are responsible for specific biological phenomenon through the integration of the modification and the associated targets of miRNAs.

Epigenetic regulation involving DNA methylation is a heritable and enzyme-induced modification in human, which modulate the expression of target mRNA without direct changing of the DNA sequences. Epigenetic modification of mRNA CpG islands has been widely reported to down-regulate the target mRNA expression in cancer-related malignant phenotypes [5, 6]. The hyper-methylation of promoter CpG island affect not only tumor suppressive mRNAs, but also tumor suppressive miRNAs. The hyper-methylation in the CpG islands of miRNA promoter can silence the expression of tumor-suppressive miRNAs or drug sensitizing miRNAs, resulting in oncogenic or chemo-resistant phenotypes in cancers. Some tumor suppressive miRNAs such as miR-34a and miR-375 have been reported to be silenced by the hyper-methylation of their promoter regions and play important roles in the related cancers [7, 8].

In the present study, we found that the promoter region of miR-129-5p was hyper-methylated in gastric cancer MDR cell lines. Furthermore, the function of miR-129-5p methylation in cancer drug resistance and the potential mechanisms were explored. Moreover, members of the MDR associated ABC transporters were found to be direct targets of miR-129-5p, indicating an important role of the methylation of this miRNA in modulating MDR in gastric cancer.

## RESULTS

### MiR-129-5p was hypo-methylated in gastric cancer MDR cell lines after 5-AZA-dC treatment

**Figure 1:**
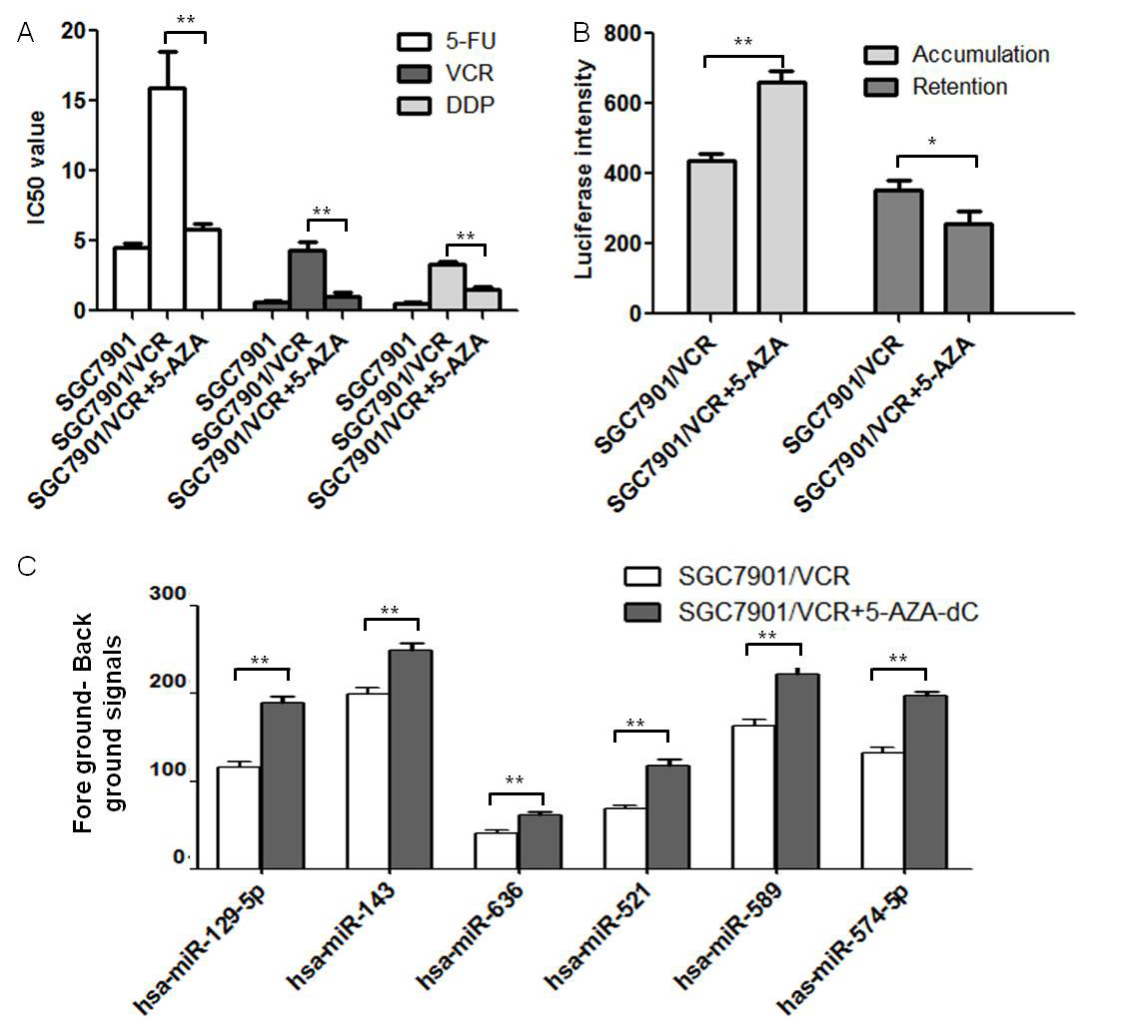
MiR-129-5p was hypo-methylated in gastric cancer MDR cell lines after 5-AZA-dC treatment A. IC50 values of cells to 5-FU, VCR and DDP calculated from MTT assays showing the effects of 5-AZA-dC on MDR in SGC7901/VCR cells compared with the parent SGC7901 cells or SGC7901/VCR cells. Each experiment was independently repeated at least 3 times. Error bars correspond to the mean ± SD. (**p<0.01). B. ADR accumulation and retention values were tested using Flow Cytometer analysis and the luciferase intensities of accumulation and retention on 5-AZA-dC treated or untreated SGC7901/VCR cells were indicated. Each experiment was independently repeated at least 3 times. Error bars correspond to the mean ± SD. (**p<0.01, *p<0.05). C. Fore ground minus back ground signals from microarray analysis. Signals of six miRNAs were found to be significantly increased in 5-AZA-dC treated SGC7901/VCR cells. Error bars correspond to the mean ± SD. (**p<0.01).

It was previously found that the methylation of miRNA promoters might silence the target miRNAs and result in the development of cancer diseases [9]. In order to test the effects of hypo-methylation in gastric cancer MDR cell lines, we treated the vincristine resistant gastric cancer MDR cell line SGC7901/VCR with 2μM de-methylation reagent 5-AZA-dC. The SGC7901/VCR cell line was established using Vincristine with consistent small doses' treatment in our lab. During the induction, the cross resistant of the cell line with other chemotherapeutic drugs (5-FU and DDP) occur, which was called “multi-drug resistance”. Using MTT, we found the IC50 values to drugs 5-fluorouracil (5-FU), Vincristine (VCR) and Cisplatin (DDP) were decreased after 5-AZA-dC treatment (Figure 1A). ADR accumulation and retention assay showed that the accumulation of 5-AZA-dC treated cells was increased and the retention was decreased compared with untreated cells (Figure 1B), indicating that the drug resistant abilities of SGC7901/VCR cells were decreased after 5-AZA-dC treatment.

To further explore the miRNA molecules that were modulated epigenetically in the above process, we performed microarray analysis using 2μM 5-AZA-dC treated MDR gastric cancer cells and the parent SGC7901/VCR cell line was used as a negative control. The decreased miRNAs in 5-AZA-dC treated SGC7901/VCR cells were then calculated using ANOVO analysis. 6 miRNAs were found to be significantly down regulated in 5-AZA-dC treated SGC7901/VCR cells compared with SGC7901/VCR cell line. Among them, miR-129-5p was chosen for further demonstration using BSP analysis because it had a most significant difference from the control cell line (1.63 fold increase in 5-AZA treated SGC7901/VCR cells). (Figure 1C).

### MiR-129-5p was hyper-methylated and down-regulated in MDR gastric cancer cell lines

**Figure 2:**
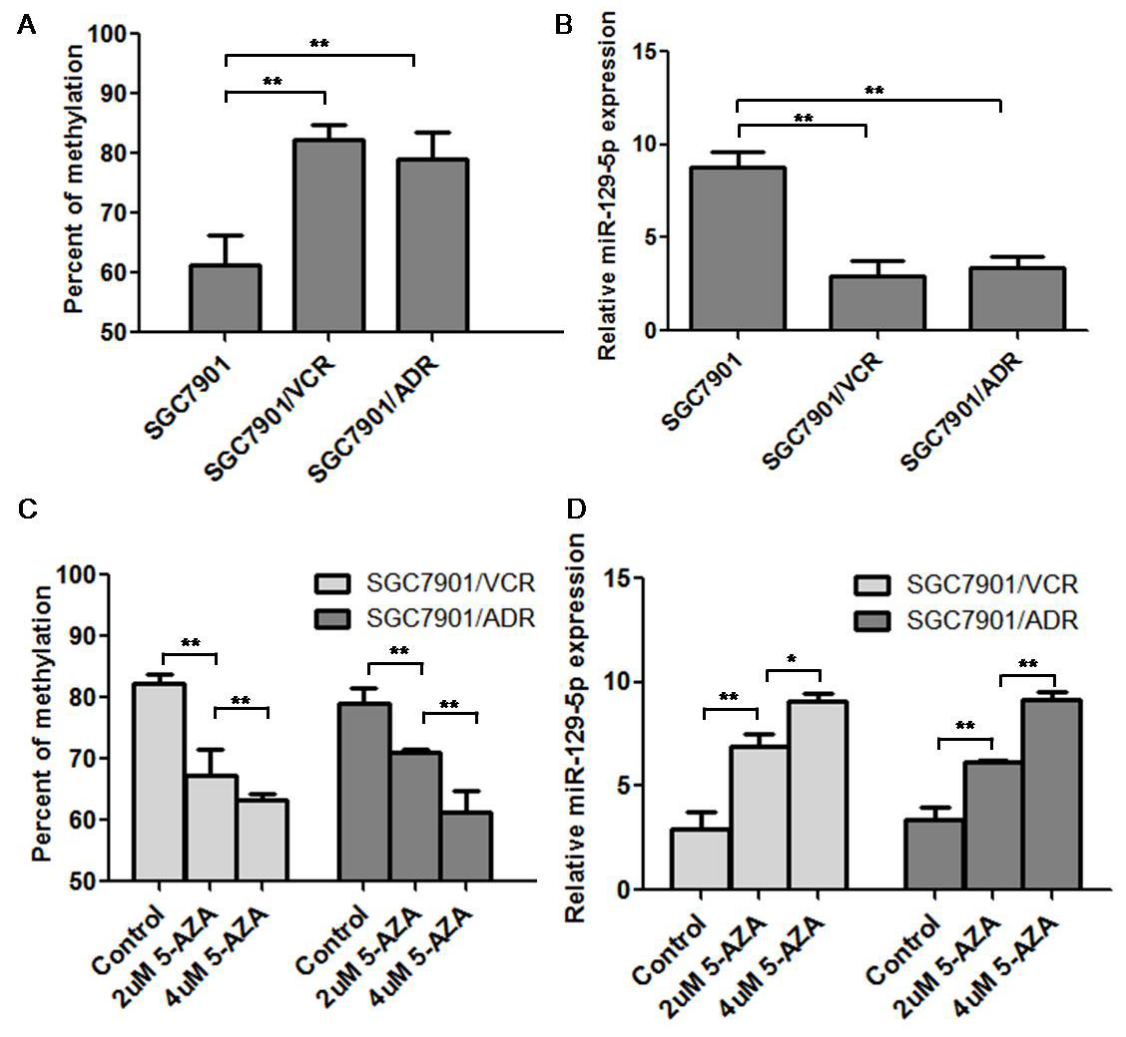
MiR-129-5p was hyper-methylated and was down-regulated in MDR gastric cancer cell lines A. The methylation status of miR-129-5p in SGC7901/VCR, SGC7901/ADR and SGC7901 cell lines was determined by BSP assay. Shown was the methylation percent of in tested cells. Error bars correspond to the mean ± SD. (**p<0.01, *p<0.05). B. Real-time PCR was used to test the miR-129-5p expression in SGC7901/VCR, SGC7901/ADR and SGC7901 cell lines and the relative expression in these cells was indicated. Each experiment was independently repeated at least 3 times. Error bars correspond to the mean ± SD. (**p<0.01). C. The methylation status of miR-129-5p in SGC7901/VCR, SGC7901/ADR cell lines after the treatment of 5-AZA-dC (2μM and 4μM) was determined by BSP assay and the methylation percent was analyzed. Error bars correspond to the mean ± SD. (**p<0.01, *p<0.05). D. MiR-129-5p expression in SGC7901/VCR and SGC7901/ADR cell lines after treatment of 2μM and 4μM 5-AZA-dC was determined by real-time PCR and the relative miR-129-5p expression value was indicated. Each experiment was independently repeated at least 3 times. Error bars correspond to the mean ± SD. (**p<0.01).

The degree of miR-129-5p methylation was further demonstrated using BSP analysis. As shown in Figure 2A, the methylation degree of miR-129-5p CpG islands were increased to 70~80% in the MDR cell lines SGC7901/VCR and SGC7901/ADR compared with the parent SGC7901 cell line which was only 61% methylated. Real-time PCR showed that the expression of miR-129-5p was significantly down regulated in SGC7901/VCR and SGC7901/ADR cell lines compared with the parent SGC7901 cells (Figure 2B). Under the treatment of serial diluted 5-AZA-dC (2μM and 4μM 5-AZA-dC), the methylation percent of MDR SGC7901/VCR and SGC7901/ADR cells were gradually decreased in 2μM and 4μM 5-AZA-dC treated groups compared with the control group (Figure 2C); while the relative miR-129-5p expression was gradually increased in 2μM and 4μM groups compared with the control group (Figure 2D).

### MiR-129-5p modulates multi-drug resistance in gastric cancer cell lines

**Figure 3:**
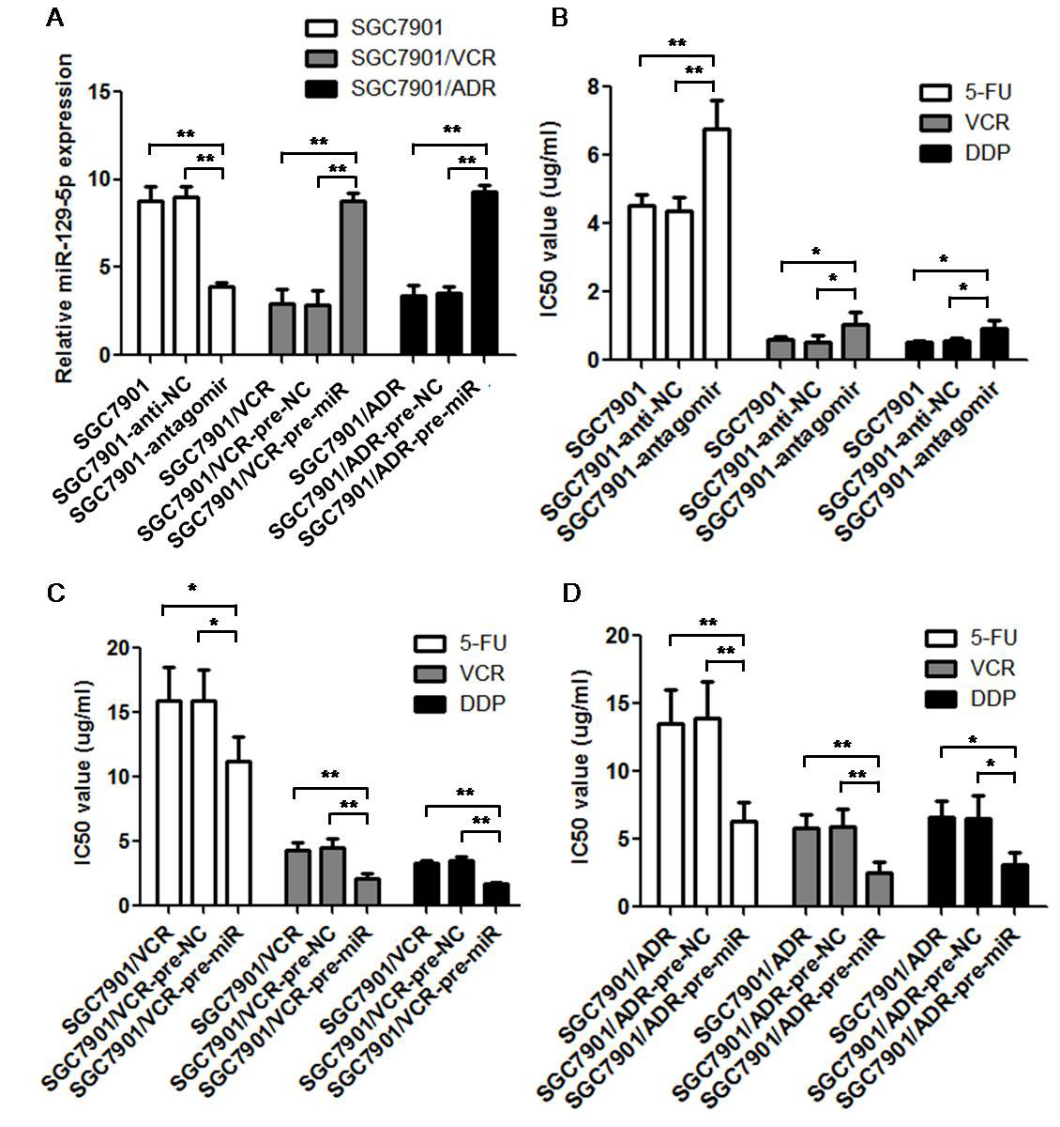
MiR-129-5p modulate multi-drug resistance in gastric cancer cell lines Transient transfection efficiency was determined by real-time PCR. The relative expression of miR-129-5p in antagomirs transfected SGC7901 cells, pre-miRs transfected SGC7901/VCR and SGC7901/ADR cells compared with the parent cell lines and the negative controls (pre-NC or anti-NC) transfected cells was indicated. Each experiment was independently repeated at least 3 times. Error bars correspond to the mean ± SD. (**p<0.01). B. C and D. IC50 values of cells to 5-FU, VCR and DDP calculated from MTT assays showing the effects of miR-129-5p antagomirs on MDR in SGC7901 cells (B), miR-129-5p pre-miRs on MDR in SGC7901/VCR cells (C), and miR-129-5p pre-miRs on MDR in SGC7901/ADR cells (D) compared with the parent cells or the negative control (NC) transfected cells. Each experiment was independently repeated at least 3 times. Error bars correspond to the mean ± SD. (**p<0.01, *p<0.05).

To test the exact function of miR-129-5p in the MDR of gastric cancer cells, the SGC7901 cells were transfected with miR-129-5p antagomir, SGC7901/VCR and SGC7901/ADR cells were transfected with miR-129-5p pre-miRs. The transfection efficiency was evaluated using real-time PCR as shown in Figure 3A. Then the IC50 values were tested using these cells by MTT assay. As shown in Figure 3B, miR-129-5p antagomir transfection in SGC7901 cells significantly increased the IC50 values of the chemotherapeutic drugs (5-FU, VCR and DDP) compared with both negative control transfection (SGC7901-anti-NC) and the parent SGC7901 cells. While transient transfection of pre-miR-129-5p into the SGC7901/VCR and SGC7901/ADR MDR cell lines significantly decreased the IC 50 values of the chemotherapeutic drugs compared with pre-NC transfection and the parent MDR cells (Figure 3C and 3D).

To avoid the cell line-specific effect, we performed chemo sensitivity assay again in another two gastric cancer cell lines (MKN45 and MKN28) using transient transfection of miR-129-5p. The transfection efficiency of both pre-miR-129-5p and antagomirs in MKN45 and MKN28 cells were validated using real-time PCR as shown in supplementary figures 1A and 1C. Transient transfection of pre-miR-129-5p in gastric cancer cell lines also decreased the IC 50 values of the chemotherapeutic drugs compared with the negative control groups; while miR-129-5p antagomir had an opposite effect as indicated in Supplementary Figures 1B and 1D.

### Members of the ABC transporter family were direct targets of miR-129-5p

**Figure 4:**
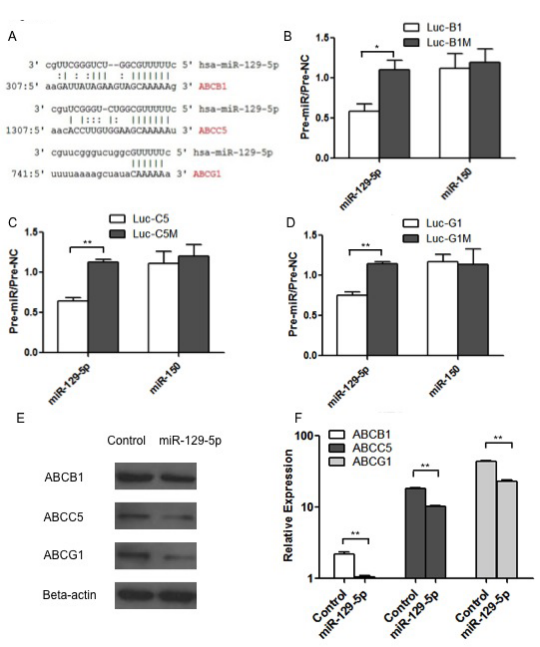
Members of the ABC transporter family were direct targets of miR-129-5p A. Bioinformatics analysis showing the conserved putativebinding sites for miR-129-5p in ABCB1, ABCC5 and ABCG1. B. C and D. Luciferase assays were performed with Luc-B1(B), Luc-C5(C),Luc-G1(D) and the mutant constructLuc-B1M(B), Luc-C5M(C), Luc-G1M(D). Bars indicate the ratio of firefly luciferase (normalized to Renilla luciferase) activity measured following transfection with miR-129-5p or miR-150 pre-miRNA compared with the activity measured following transfection with the pre-miR-control (pre-NC) for the same construct. Each experiment was independently repeated at least 3 times. Error bars correspond to the mean ± SD. (**p<0.01, *p<0.05). E and F. Western blot (E) or Real-time PCR (F) showing the changes in the protein levels (E) or RNA levels (F) of ABCB1, ABCC5 and ABCG1 after transient transfection of pre-miR-129-5p compared with the negative controls (Control) respectively. Error bars correspond to the mean ± SD. (**p<0.01).

In order to explore the direct targets of miR-129-5p in modulating MDR in gastric cancer, bioinformatics analysis was used. In silico analysis using MiRanda software (http://www.microrna.org/microrna/home.do) showed that the 3′-UTR of three members of ABC transporters (ABCB1, ABCC5 and ABCG1) contains conserved putative target sites separately for miR-129-5p as shown in Figure 4A. To further validate these target sites, the 3′-UTRs of human ABCB1, ABCC5 and ABCG1 were inserted downstream of the luciferase gene in the pGL3-Control vector, providing with Luc-ABCB1 (B1), Luc-ABCC5 (C5) and Luc-ABCG1 (G1), which were referred to Luc-B1, Luc-C5 and Luc-G1 and the mutant constructs (Luc-B1M, Luc-C5M and Luc-G1M) in Figure 4B, 4C and 4D.

Report gene assay showed that transfecting HEK-293 cells with miR-129-5p significantly decreased the expression of Luc-B1, Luc-C5 and Luc-G1, while displaying no effect on Luc-B1M, Luc-C5M and Luc-G1M expression (Figure 4B, 4C and 4D). In contrast, miR-150 did not exhibit any effect on the expression of Luc-B1, Luc-C5 or Luc-G1 (Figure 4B, 4C and 4D), in accordance with the fact that the three 3′-UTR contains no miR-150 target sites.

To determine the down-regulating effects of miR-129-5p at the protein level, we performed western blotting assays using ABCB1, ABCC5 and ABCG1 antibodies. As indicated in Figure 4E, the transient transfection of SGC7901 cells with pre-miR-129-5p decreased ABCB1, ABCC5 and ABCG1 protein levels, which suggests that miR-129-5p miRNAs regulate ABCB1, ABCC5 and ABCG1 expression *in vivo* at the post-transcriptional level. The RNA levels of ABCB1, ABCC5 and ABCG1 were also determined by real-time PCR as shown in Figure 4F.

### Anti-miR-129-5p modulated MDR in tumor-bearing nude mice

**Figure 5:**
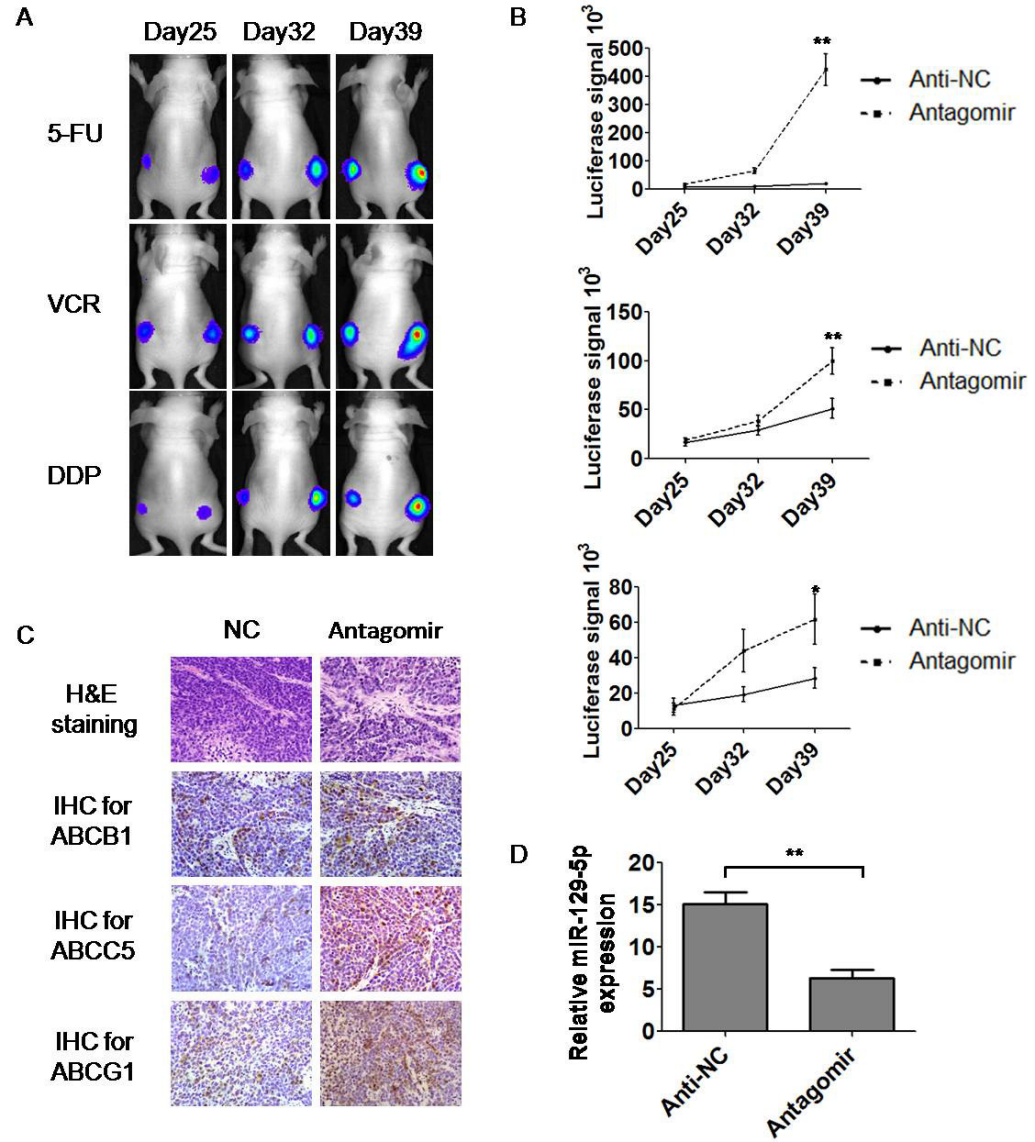
MiR-129-5p antagomirs modulated MDR in tumor-bearing nude mice A and B. Representative images showing the luciferase signals of tumors treated or untreated with miR-129-5p on the back of nude mice on the day 25, 32 and 39 under the treatment of 5-FU, VCR and DDP (A). Comparison of luciferase signals on the day 39 was analyzed and indicated (B). Error bars correspond to the mean ± SD. (**p<0.01, *p<0.05). C. Representative images show H&E staining of the miR-129-5p treated or untreated tumors (×400) generated in nude mice and immunohistochemistry staining using ABCB1, ABCC5 and ABCG1 antibodies on these tumors (×400). D. Relative miR-129-5p expression in tumors generated from nude mice was determined by real-time PCR. Expression of miR-129-5p was significantly down regulated in antagomir injected tumors compared with anti-NC injected ones. Error bars correspond to the mean ± SD. (**p<0.01).

To test the *in vivo* relationships between miR-129-5p and MDR, we injected BALB/C nude mice subcutaneously with gastric cancer cells. To facilitate the detection of tumor size, a luciferase-labeled SGC7901-Luc cell line established in our lab was used for injection. Six mice were injected for each group. When the tumors reach around 5mm in size, the LNA (Lock Nucleic Acid) decorated antagomir of miR-129-5p was injected into the tumors 6–8 sites of the tumors at the concentration of 110 μg/kg/day. Three days later, the mice were intraperitoneally injected using PBS with chemotherapeutic drugs 5-FU, VCR and DDP two injections a week. The luciferase signals of the tumors were tested every week under the detection of IVIS 100 system. The luciferase signals of tumors on the day 25, 32 and 39 were shown in Figure 5A and the analysis of signals were shown in Figure 5B. Eight weeks after the first injection, the nude mice were killed, and part of the tumors from the nude mice were harvested, fixed and embedded in paraffin for further staining. The formation of tumors was confirmed by H&E staining (Figure 5C). Immunohistochemistry showed that the expression of the ABC transporters ABCB1, ABCC5 and ABCG1 were increased in miR-129-5p antagomir treated tumors compared with negative control (anti-NC) treated tumors as indicated in Figure 5C. Real-time PCR was further used to determine the expression of miR-129-5p in the generated tumors. The results showed that tumors with miR-129-5p antagomir treatment had a decreased expression of miR-129-5p compared with NC treated tumors as shown in Figure 5D.To test whether treatment of antagomir alone would have an impact on tumor growth in the absence of drug treatment, we tested the effect of antagomir group compared with the NC group with or without the treatment of the anti-cancer drug 5-FU. It is interesting that on the day 25 after injection, the antagomir alone group grew still faster than the NC group in both 5-FU treated or untreated mice, as shown in Supplementary Figure 2. Both the antagomir alone group and the NC group grew bigger in 5-FU untreated mice, however, the NC group under PBS treatment grew even bigger. These results suggested that although miR-129-5p antagomir alone had the ability to trigger tumor growth in nude mice, it promoted growth and drug-resistance even more under the treatment of anti-cancer drugs. The present study also indicated that miR-129-5p is a candidate in the future study for the therapeutics of gastric cancer drug-resistance.

## DISCUSSION

The CpG island methylation of tumor suppressive miRNAs was widely discovered in various cancers all over the world. The methylation modulated silencing of miRNAs was found to play significant roles in the malignant phenotypes of cancer development including cell proliferation, migration and invasion, apoptosis, cell cycle as well as MDR. For example, methylation of miR-203 was found to be apparent in pre-cancerous lesions. Ectopic expression of miR-203 was found to decrease both the proliferation rate and anchorage independent growth in cervical cancer cells [10]. Hyper-methylation mediated the silencing of miR-124, which was a frequent event in pancreatic duct adenocarcinoma. Functional studies showed that miR-124 inhibited cell proliferation, invasion and metastasis by targeting Rac1 [11]. MiR-34b is silenced in human prostate cancer and the mechanism is through CpG hyper-methylation. MiR-34b directly targeted methyl-transferases and de-acetylases, resulting in a positive feedback loop inducing partial de-methylation and active chromatin modifications. Functionally, miR-34b inhibited cell proliferation, colony formation, migration/invasion, and triggered G(0)/G(1) cell-cycle arrest and apoptosis by directly targeting the Akt and its downstream proliferative genes [12].

MiR-129 was firstly found to be down regulated in undifferentiated gastric cancer tissues [13]. Later on, miR-129 was found to exert growth inhibition and induce cell death upon over-expression in bladder carcinoma, hepatocellular carcinoma, esophageal carcinoma and gastric cancer [14-17]. Methylation of miR-129-2 CpG Island was frequently observed in various cancers such as colorectal cancer, esophageal squamous cell carcinoma, hepatocellular carcinoma and gastric cancer [18-23]. In endometrial cancer, reactivation of miR-129-2 in cancer cells by pharmacologic induction of histone acetylation and DNA de-methylation resulted in decreased SOX4 expression. In addition, restoration of miR-129-2 by cell transfection also led to decreased SOX4 expression and reduced proliferation of cancer cells [24]. In colorectal cancer, ectopic expression of miR-129 promoted apoptosis, inhibited cell proliferation and caused cell-cycle arrest by suppressing a key anti-apoptotic protein, B-cell lymphoma 2 (BCL2) [25].

In gastric cancer, the expression of miR-34b and miR-129-3p was down regulated by DNA hyper-methylation in primary gastric cancers, and the low expression was associated with poor clinic-pathological features [23]. The epigenetic repression of miR-129-2 leads to overexpression of SOX4 and the up-regulation of SOX4 was inversely associated with the epigenetic silencing of miR-129-2 in gastric cancer [24]. Although previous studies found the promoter of miR-129-2 was hyper-methylated in gastric cancer,in the present study, we found the CpG Island of miR-129-5p was even more hyper-methylated in gastric cancer multi-drug resistant cell lines compared with the parent gastric cancer cell lines. De-methylation treatment of SGC7901/VCR cells using 5-AZA-dC found an increased miR-129-5pexpression. Furthermore, gain of function assays using pre-miRNAs indicated that the over-expressed miR-129-5p repressed MDR in both VCR and ADR resistant cell lines. Loss of function assays using antagomirs, which mimics the methylation modulated silence of miR-129-5p expression promoted MDR in gastric cancer SGC7901 cell line. Our findings indicate that the hyper-methylated miR-129-5p plays a pro-drug-resistant function in gastric cancer multi-drug resistant cells and that the hypo-methylation of miR-129-5p might reverse MDR in gastric cancer cells.

Some studies have reported different results regarding the expression of miR-129 in cancers. For example, miR-129 was found to be highly expressed in oral squamous cell carcinoma [26], esophageal cancer [27], malignantly transformed human bronchial epithelial cells [28], and retinoblastoma [29] compared with the normal tissues. However, in these reports, the potential functions or mechanisms of miR-129 in cancers were not further explored or studied. This might be because of the differential expression of the cancer genome among various parts of the body. Our present study did not focus on the comparison of miR-129-5p expression in cancer with normal cells, but focused on the function of hyper-methylated miR-129-5p in multi-drug resistant cells compared with the parental cancer cells. The present findings demonstrated that multi-drug resistant of cancer cells could also be modulated by the hyper-methylation of miRNA CpG island.

The ABC transporter family was known to have at least 48 members identified in humans and 12 of them were recognized to be putative drug transporters [30, 31]. If patients with tumors have a higher expression of various ABC transporter pumps, they usually do not respond to chemotherapy because the ABC transporters located on the cytoplasm side of the resistant cell will efflux chemotherapeutic drugs outside the tumor cells, leading to chemo-resistance. In the present study, we found three members of MDR related ABC transporters (ABCB1, ABCC5 and ABCG1)were targeted by miR-129-5p, which was a hyper-methylated miRNA in gastric cancer MDR cell lines. Once the miR-129-5p were hyper-methylated and silenced in gastric cancer cells, the expression of MDR related ABC transporters (ABCB1, ABCC5 and ABCG1) would increase and directly lead to a MDR phenotype.

## MATERIALS AND METHODS

### Ethics statement

In the animal experiments, all procedures for animal experimentation were performed in accordance with the Institutional Animal Care and Use Committee guidelines of the Experimental Animal center of the Fourth Military Medical University. The approval ID for using the animals was No.12566 from Experimental Animal Center of the Fourth Military Medical University.

### Cell culture and 5-Aza-dC treatment

The human gastric adenocarcinoma cell line SGC7901 was obtained from the Academy of Military Medical Science (Shanghai, China). Gastric cancer vincristine resistant SGC7901/VCR cell line and doxorubicin resistant cell line SGC7901/ADR were constructed in our institute [32, 33]. All the cell lines were preserved in our institute. SGC7901-Luc cells stably expressing firefly luciferase were generated and preserved in our lab. All of the cells were grown in RPMI1640 (Invitrogen, Carlsbad, CA, USA) supplemented with 10% heat-inactivated fetal calf serum (FCS) at 37°C with 5% CO2 in a humidified incubator (Forma Scientific, Marietta, OH, USA). For 5-Aza-dC treatment, cells were cultured with 5-Aza-dC (2.5μM) for 48h, and then were harvested for the analysis of microarray or for the detection of miRNA expression.

### Transient transfection

Transient transfection was performed with siPORT™ NeoFX™ Transfection Agent (AM4511, Applied Biosystems, USA) according to the manufacturer's recommendations. The precursors (pre-miRs) and antagomirs (anti-miRs) of the miR-129-5p were obtained from Applied Biosystems (Invitrogen, Carlsbad, CA, USA), supplied with negative control (NC) miRNAs (pre-NC or anti-NC). Cells were harvested 48–72 h after transient transfection.

### Microarray

Microarray analysis was performed using a service provider (Kangcheng Biology Corporation) in 2 to 5 μg of total RNA from SGC7901/VCR cells and SGC7901/VCR cells treated with 2μM 5-AZA-dC. Total RNA was harvested using TRIzol (Invitrogen) and RNeasy mini kit (QIAGEN) according to manufacturer's instructions. After having passed RNA measurement on the Nanodrop instrument, the samples are labeled using the miRCURY™ Hy3™/Hy5™ Power labeling kit and hybridized on the miRCURY™ LNA Array (v.11.0). The samples were hybridized on a hybridization station following the scheme you outlined in the sample submission. Scanning is performed with the Axon GenePix 4000B microarray scanner. GenePix pro V6.0 is used to read the raw intensity of the image.

### Intracellular ADR intensity analysis

The fluorescence intensity of intracellular ADR was determined using flow cytometer analysis as described before [34]. Specifically, log phase cells were plated in six-well plates (1×10^6^ cells/well) over night and were exposed in ADR chemotherapeutic drugs to a final concentration of 1.3 g/ml. Cells were then cultured for 1 h and harvested to test ADR accumulation or continuously cultured in drug-free medium for another 2 hs, following by the detection of ADR retention. Finally, cells were washed twice with cold PBS and the mean fluorescence intensity of intracellular ADR was detected using flow cytometer analysis. The ADR-releasing index of cells was calculated using the formula: releasing index = (accumulation value - retention value)/accumulation value.

### *In vitro* drug sensitivity assay

A 3-(4,5-Dimethylthiazol-2-yl)-2,5-diphenyl-tetrazolium bromide (MTT) assay was performed to evaluate cell growth ability as described previously [35]. 5 × 10^3^ cells transiently transfected with pre-miR-129-5p, miR-129-5p antagomir or negative controls were collected in 200 μl of complete medium, plated in 96-well plates and incubated under normal conditions. Cultures were assayed at 1, 2, 3, 4 and 5 days, and 490 nm absorbance (A490) was read on a micro plate reader (168–1000 Model 680, Bio-Rad, Hercules, USA). The inhibition rates and IC50 values were then calculated. Each experiment was performed in triplicate and repeated for three times.

### *In vivo* drug sensitivity assay

BALB/C nude mice at 4-6 weeks of age were handled using best humane practices and cared for in accordance with NIH Animal Care Institutional Guidelines in the Experimental Animal Center of the Fourth Military Medical University (Xi'an, Shaanxi Province, P. R. China). 1 × 10^7^ SGC7901-Luc cells in 0.2 ml cell culture medium were injected subcutaneously into the left and right upper back at a single site. Six mice were injected for each group. When the mean tumor volume reached 100–200mm^3^, mice were randomized to start the injection two consecutive days with 10 μl PBS containing miR-129-5p antagomir or negative control RNAs at 6–8 sites of the tumors (110 ug/kg/day). Three days later, the mice were intraperitoneally injected with PBS containing VCR (0.3mg/kg), 5-FU (5mg/kg) or DDP (2.5mg/kg) two injections per week. The mice were injected with 100mg/kg D-luciferin intraperitoneally five minutes before imaging. Bioluminescent signals were detected on the 7th, 14th and 21th day after injection of the antagomir using the IVIS 100 Imaging System (Xenogen, Hopkinton, MA). Nude mice were killed 30 days after first injection. Their subcutaneous tumors were harvested and fixed in 10% formalin before paraffin embedding, then sectioned and stained in H&E.

### Report gene assay

For reporter gene assay, cells were plated in 12-well plates and were transfected with 2 μg of the target genes' 3′-UTR luciferase reporter plasmids (S) or the target gene 3′-UTR mutant (M) and the empty pGL3-Control vector (Promega Biotech Co., Ltd, Beijing, China) using lipofectamine 2000 (Invitrogen, Carlsbad, CA, USA). Cells were also co-transfected with the pre-miRNAs (150 nM, Applied Biosystems, Invitrogen, Carlsbad, CA, USA). Assays were performed 24 h after transfection using the Dual Luciferase Reporter Assay system (Promega Biotech Co., Ltd, Beijing, China). Firefly luciferase activities were normalized to Renilla luciferase activities. A microRNA precursor molecule control from Applied Biosystems (Invitrogen, Carlsbad, CA, USA) was used as one of the negative miRNA controls and was referred to as pre-miR-NC. All experiments were performed in triplicate.

### Bisulfite sequencing PCR

For bisulfite sequencing PCR (BSP), Genomic DNA was extracted from cells using TRIZOL (Invitrogen, Carlsbad, CA, USA), and was then subjected to bisulfite conversion using the EZ DNA Methylation-Gold Kit (Zymo Research Corporation, Orange, CA) according to the manufacturer's instructions. The bisulfite-converted genomic DNA was used for the methylation analysis of miR-129-5p with the predicted methylation primers, which were designed according to the online primers program “MethPrimer” (http://www.uro-gene.org/methprimer/). The primers used for MSP are as follows: M-miR-129–F 5′ GTTGGGGAGATTTAGTTTGTT 3′ M-miR-129–R 5′ CCTACTCCAATTCCCCCTATAATAC 3′

The amplified fragments were cloned into the pGEMT Easy vector (Promega, Madison, WI), and five to ten clones were randomly selected for bisulfite sequencing.

### RNA extraction and Real-time PCR

Total RNA from cells or tumors from mice was extracted using TRIZOL (Invitrogen, Carlsbad, CA, USA) with RNase-free DNase. Reverse transcription was performed according to the manufacturer's instructions (D350A, TaKaRa Biotechnology (DALIAN), Co., Ltd, DaLian, Liaoning Province, China). qRT-PCR was performed to determine the expression levels of each miRNA using the exact sequences (U to T) of these miRNAs as the forward primers and the unique q-PCR primer from the cDNA Synthesis Kit as the reverse primer. U6 was used as an internal control, and each plate contained one cDNA sample for each primer as a calibration sample. All experiments were performed in triplicate.

### Western Blot

To determine the levels of protein expression, log phase cells were harvested from 90 mm culture plates, lysed in RIPA lysis buffer (150 mMNaCl, 50 mMTris–HCl (pH 8.0), 0.1% SDS, 2 mM EDTA, 1 mM PMSF, 1% NP40, 5 ug/ml aprotinin, and 1 ug/ml leupeptin) on ice, and then centrifuged at 12000 rpm for 10 min. Total proteins were resolved by 12% SDS-PAGE (Bio-Rad Laboratories, Inc, Hercules, CA, USA) and blotted onto nitrocellulose membranes (Amersham Biosciences Corp., Pittsburgh, PA, USA). Membranes were blocked with 10% non-fat milk powder at room temperature for 2 h and incubated overnight with primary antibodies: anti-ABCB1 (1:1000; Abcam plc. Cambridge, MA, USA), anti-ABCC5 (1:2000; Abcam plc. Cambridge, MA, USA), anti-ABCG1 (1:1500; Abcam plc. Cambridge, MA, USA), or anti-β-actin antibody (1:2000; Sigma-Aldrich Co. Louis, MO, USA). After three 5 min washes in Triethanolamine-Buffered Saline Solution with 0.1% Tween-20 (TBS-T), membranes were incubated with horseradish peroxidase (HRP) conjugated secondary antibodies (1:2000; santa cruz biotechnology, inc.Dallas, TX, USA) for 4 h at room temperature and then washed again in TBS-T and visualized with an enhanced chemi-luminescence kit (ECL-kit, Santa cruz Biotechnology, Inc. Dallas, TX, USA). All experiments were performed in triplicate.

### Immunohistochemistry

Tumor samples generated from nude mice were taken for paraffin embedding, and serial 4μm sections were used for immunohistochemistry staining. Deparaffinized and rehydrated sections were washed in fresh water for 10 min. Heat-induced antigen retrieval was performed for 20 min at 95°C with 10 mM citrate sodium buffer (PH 6.0). After the sections were cooled at room temperature for 40 min, they were blocked in 3% hydrogen peroxide for 20 min and then in normal goat serum confining liquid for 40 min. After this, the sections were allowed to react over night at 4°C with primary antibody for ABCB1, ABCC5 and ABCG1 (Santa Cruz). After rewarming for 40 min, the slides were reacted with second antibodies (Zhongshan Goldenbridge Biotechnology CO. LTD) for another 40 min at room temperature. Then the products were developed with 3,3′-diaminobenzidine and counterstained with haematoxylin.

### Statistical analysis

Continuous variables were compared by Student's t test or an ANOVA test. If the test result of the homogeneity of variances between the groups was significant, the Mann-Whitney test was appropriately adopted.In samples with small size (n<30) and with non-normal distribution and/or elevated dispersion, we also used non-parametric statistics. All statistical analyses were conducted using SPSS software, version 14.0 (Chicago, Illinois, USA).

## CONCLUSION

In conclusion, the present study demonstrated that miR-129-5p plays an important role in antagonizing MDR in gastric cancer cell lines by directly targeting and inhibiting three MDR-related ABC transporters--ABCB1, ABCC5 and ABCG1. The hyper-methylation of miR-129-5p CpG island in gastric cancer MDR cells leads to an increased expression of these ABC transporters, which resulted in a direct drug resistance. Thus, our findings demonstrated miR-129-5p is significant in modulating MDR in gastric cancer and that targeting miR-129-5p might be a potential therapeutic strategy in gastric cancer gene therapies.

## SUPPLEMENTARY MATERIAL AND FIGURES


