# Supplementary material for: Methylation of miR-129-5p CpG island modulates multi-drug resistance in gastric cancer by targeting ABC transporters
Source: Oncotarget. 2014 Oct 18;5(22):11552–63. doi: 10.18632/oncotarget.2594 (PMC4294356; doi:10.18632/oncotarget.2594)
Supplement: Supplementary file 1 [file oncotarget-05-11552-s001.pdf]

Methylation of miR-129-5p CpG island modulates multi-drug resistance in gastric cancer by targeting ABC transporters

Supplementary Material

Supplementary Figure 1

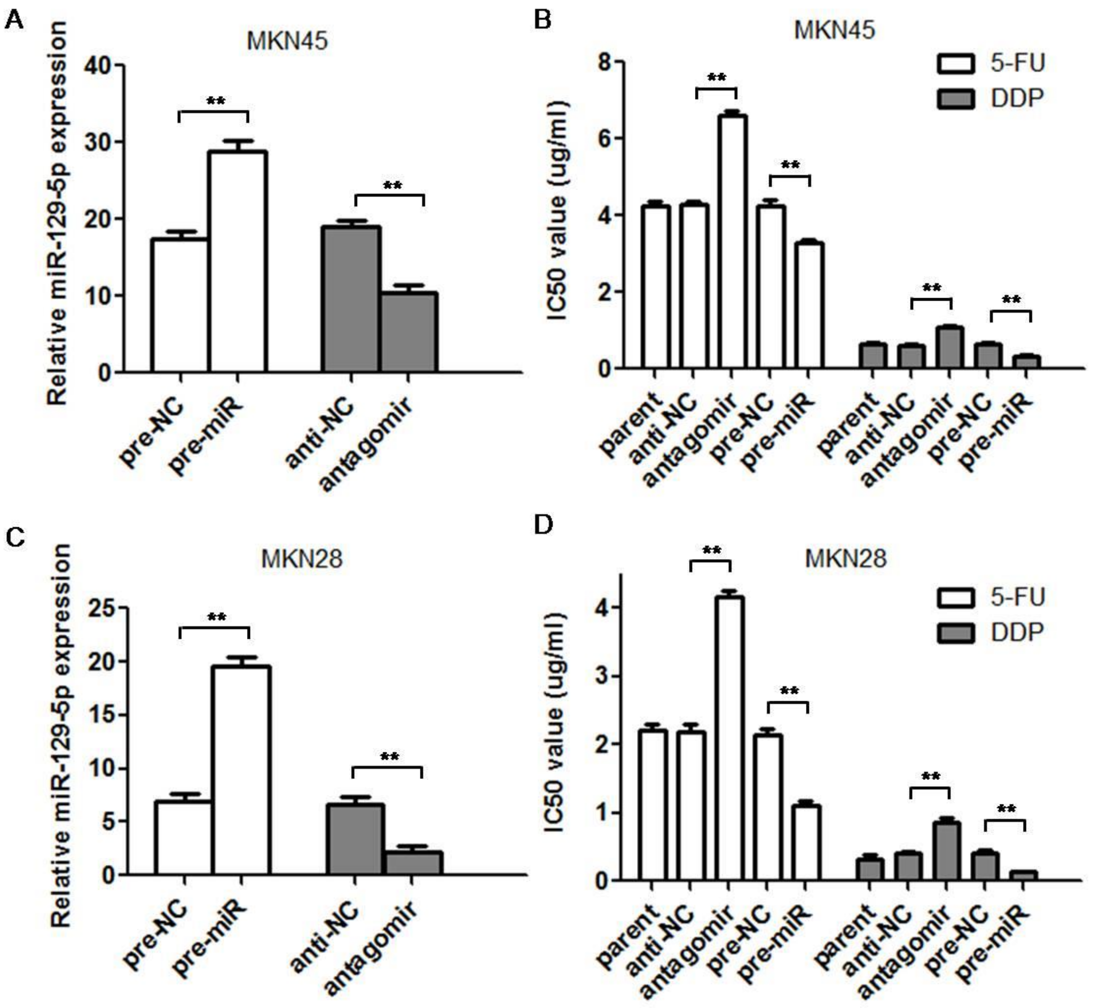

Supplementary Figure 1: A&C Real-time PCR was used to test the pre-miRs or antagomirs of miR-129-5p expression in MKN45 (A) or MKN28(C) cell lines and the relative expression in these cells was indicated. Each experiment was independently repeated at least 3 times. Error bars correspond to the mean ± SD. (**$p \leq 0.01$). B&D IC50 values of cells to 5-FU and DDP calculated from MTT assays showing the effects of miR-129-5p pre-miRs or antagomirs on MDR in MKN45 (B) or MKN28 (D) cells compared with the negative controls. Each experiment was independently repeated at least 3 times. Error bars correspond to the mean ± SD. (**$p \leq 0.01$).

Supplementary Figure 2

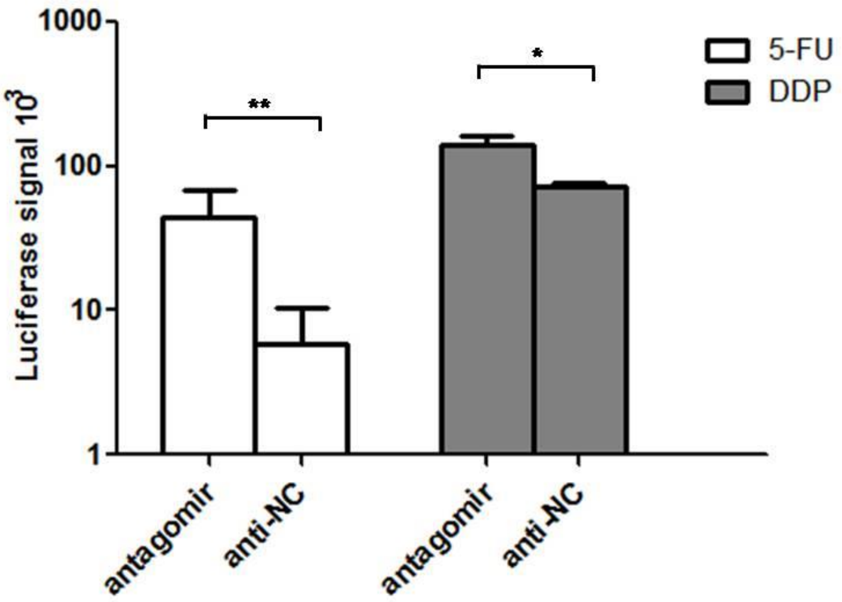

Supplementary Figure 2: Comparison of luciferase signalson the back of nude mice with or without the treatment of 5-FUwas analyzed and indicated. Nude mice were divided into two groups for the treatment of 5-FU or PBS, each group contains 5 mice, each mouse was injected for SGC7901-Luc cells into the left and right upper back at a single site. When the mean tumor volume reached 100–200mm3, mice were randomized to start the injection with miR-129-5p antagomirs or negative control RNAs at the tumors. Three days later, mice were intraperitoneally injected with PBS containing 5-FU or PBS alone two injections per week.Error bars correspond to the mean ± SD. (**p<0.01, *p<0.05)
